# Biochemical profile of *Dunaliella* isolates from different regions of Iran with a focus on pharmaceutical and nutraceutical potential applications

**DOI:** 10.1002/fsn3.4137

**Published:** 2024-04-17

**Authors:** Maryam Araj‐Shirvani, Masoud Honarvar, Mahshid Jahadi, Maryam Mizani

**Affiliations:** ^1^ Department of Food Science and Technology, Science and Research Branch Islamic Azad University Tehran Iran; ^2^ Department of Food Science and Technology, Faculty of Agriculture Isfahan (Khorasgan) Branch, Islamic Azad University Isfahan Iran

**Keywords:** carotenoid, *Dunaliella*, eicosapentaenoic acid, nutraceutical, pharmaceutical

## Abstract

This study was conducted to evaluate three species of *Dunaliella* microalgae (*Dunaliella salina*, *Dunaliella viridis*, and *Dunaliella* sp.) indigenous to Iran as new sources of natural chemical and bioactive compounds for exploring pharmaceutical and nutraceutical potential applications. The results showed that the fat, carbohydrate (mono‐ and di‐saccharide), dietary fiber, and protein content of *Dunaliella* were in the range of 13.19–25.02, 7.59–12.37, 42.10–48.82, and 17.68–22.50 (%), respectively. *Dunaliella salina* contained a pigment fraction of 11.50%, which was largely composed of carotenoid (7.41%) and chlorophyll (4.09%). Antioxidant capacity and inhibition of 2,2‐diphenyl‐1‐1‐picrylhydrazyl (DPPH) of *Dunaliella salina* were 34.54 mg/1000 g and 55.63%, respectively. The lipid profile also revealed that three isolated *Dunaliella* are remarkable sources of polyunsaturated fatty acids (25.42%–40.13%). Further, the ratios of ∑n‐3/∑n‐6 (2.79%), docosahexaenoic acid (6.15%), and eicosapentaenoic acid (11.26%) were the highest in *Dunaliella salina*. The results, thus, proved that *Dunaliella* spp., especially *Dunaliella salina* (IBRC‐M 50030), which originates from a lake in Semnan province, Iran, has potential applications in the food and pharmaceutical industries due to its appropriate biopigment, protein, lipid, antioxidant activity, long‐chain polyunsaturated fatty acids, docosahexaenoic acid, and eicosapentaenoic acid.

## Graphical abstract

The research aimed to investigate the natural chemical and bioactive compounds present in three microalgae types, namely *Dunaliella salina*, *Dunaliella viridis*, and *Dunaliella* sp., isolated from and native to three regions in Iran. The study revealed that the biomass of these microalgae contained abundant quantities of protein, dietary fiber, bioactive phenolic compounds, carotenoid, chlorophyll, and high nutritional value fats that are rich in PUFA, EPA, and DHA. Hence, these microalgae can be utilized as a natural cell factory for the biological synthesis of bioactive substances and a sustainable source of human nutritional needs, particularly *Dunaliella salina*.

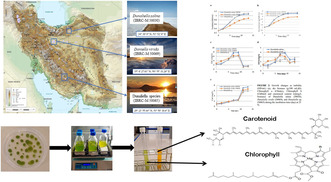

## 1 INTRODUCTION

One of the major challenges human society faces in the twenty‐first century is the need to provide nutrients for the ever‐rising population with limited natural resources (Torres‐Tiji et al., 2020). Thus, finding new and sustainable food resources is a main concern for the food industry and related organizations. Microalgae can be regarded as a stable and consumable food resource and, thus, a solution to these concerns; so the research and development needed to bring them into mass production seems to be a necessity (Ben‐Amotz, 2003; Molino et al., 2018; Polle et al., 2020).

These microscopic organisms have many benefits over their macroscopic counterparts, including better genetic manipulation, easier production processes, a high biomass yield per area, biomasses rich in bioactive materials, and the possibility of cultivation in barren lands using undrinkable water or salt water. Moreover, microalgae can be utilized in the production of many natural metabolites such as proteins, carbohydrates, lipids, and natural bioactive components with antioxidant, anti‐bacterial, anti‐fungal, anti‐inflammatory, and anti‐tumor benefits, which can be used as supplements to improve the human's diet (Gantar & Svirčev, 2008; Pal et al., 2014; Zarei et al., 2016).

Today, according to the National Center for Biotechnology Information, *Dunaliella* algae belong to the class Chlorophyceae, the order *Chlamydomonadales*, and the family *Dunaliellaceae* (Jin et al., 2003). *Dunaliella* cells are typically egg‐shaped and 4–15 μm wide and 6–25 μm long. However, depending on the growth stage and environmental conditions, the shape of the cells can change from ovoid to oval, cylindrical, pear‐shaped, and an almost spherical spindle. Moreover, these algae move using two equal‐sized flagella (Ben‐Amotz, 2003; Borowitzka & Siva, 2007). *Dunaliella* lacks a polysaccharidic cell wall; instead, these cells are covered by a layer of amorphous mucilage with variable thickness, which is called glycocalyx (Borowitzka & Siva, 2007). They have a cup‐shaped chloroplast, with a pyrenoid and a core located in the upper and colorless part of the algae (Pal et al., 2014).

Due to the massive accumulation of carotenoids, proteins, lipids, especially unsaturated fats, vitamins, and minerals, *Dunaliella* can be regarded as a high‐quality food source (Polle et al., 2020). *Dunaliella's* morphological and physiological characteristics and metabolite production depend on the growth stage, region, and local environment's cultivation conditions, such as nutritional availability, light intensity, and temperature fluctuations (Ben‐Amotz, 2003; Borowitzka & Siva, 2007; Polle et al., 2020). It is also noteworthy that *Dunaliella* is the only photosynthetic organism capable of living in habitats with a salinity of 0.5–5 M sodium chloride (Pal et al., 2014). Its simple cell structure and culture have made it a suitable organism for studying the mechanisms of resistance to environmental stress at a molecular level (Liang et al., 2020).

Bioprospecting of microalgae is defined as “the identification of economically valuable biochemical resources from algae rich in such content and enables industrial bioresource generation” (Khosravinia et al., 2023). Therefore, exploring native microalgae and assessing the competency of their bioactive metabolites and their productivity can be highly significant. Moreover, native microalgae species of all countries adapt to their environment. Environmental factors related to distribution and indigenousness play an important role in the structure and shaping of microbial communities. Studies have shown the locality's effects on morphological features and microalgae metabolite production clearly (Sharma & Rai, 2011). The effects of environmental conditions hand in hand with distribution are undeniable because these factors not only affect the microalgae's biology (asexual or sexual production, spore production potential, etc.) but also influence the composition of natural communities (Vanormelingen et al., 2007). Twelve isolated strains of microalgae were considered for high saturated fatty acid and biofuel production (Piligaev et al., 2015), naturally isolated microalgal strains were investigated for different potential SCP, *β*‐carotene, and fatty acids including *α*‐linolenic acid and linoleic acid (Dinpazhooh et al., 2022), antioxidant activity of different microalgae in Morocco (Maadane et al., 2015) and high poly unsaturated fatty acid and high eicosapentaenoic acid (EPA) of *Dunaliella salina* obtained from Bombay (India) (Bhosale et al., 2010) were produced for pharmaceutical and biotechnological applications. Morphology, physiology, and molecular approaches aiming to gain a better understanding of the specie's categorization and the diversity of Indian *Dunaliella salina* show that locality has a great impact on microalgae characteristics (Preetha et al., 2012). Therefore, the exploitation of native strains that have high compatibility with their local environmental conditions is a logical strategy to obtain species with potential uses in the production of bioactive compounds.

In this regard, research on the direct use of *Dunaliella* or the application of its metabolites in the food and pharmaceutical industries is very limited around the world, especially in Iran. Iran's climate, vast area of land, and low rainfall make it necessary to change the focus of research studies from meeting food needs from agriculture to investigating microalgae. The present research was, therefore, conducted to find out more about the biochemical composition of *Dunaliella* spp. as a microalga that is important due to its satisfactory lipid content, high protein, carotenoid, antioxidant activity for direct use, or the use of its metabolites in the food and pharmaceutical industries. The biochemical composition and growth rate of three isolated *Dunaliella* spp., separated from three geographical regions in Iran, were considered for their potential nutritional and pharmaceutical applications and for producing agricultural products under dire situations such as droughts with low environmental damage and low cost.

## 2 MATERIALS AND METHODS

### 2.1 Microalgae source

**FIGURE 1:**
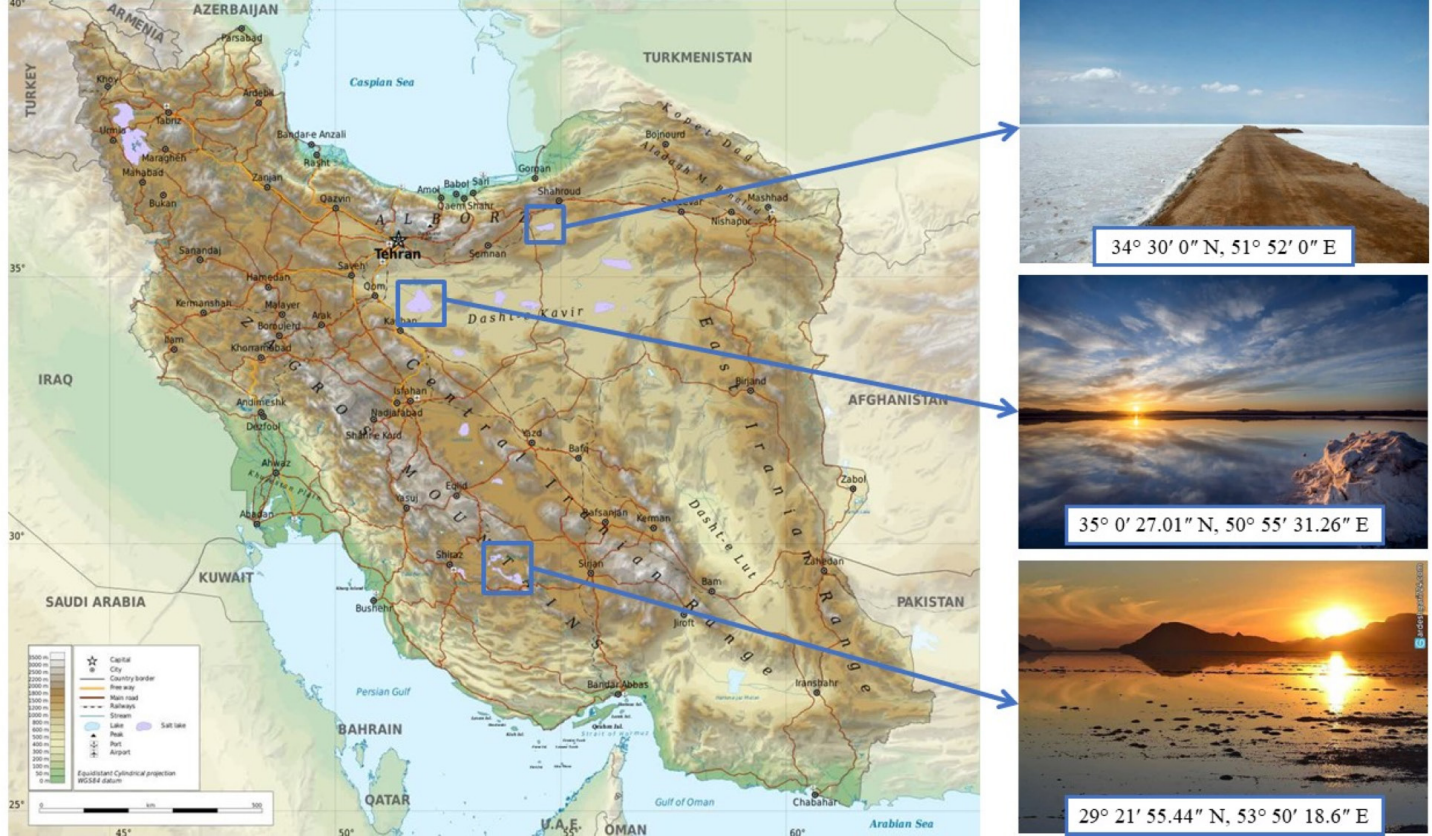
Map of the rivers showing the regions of Iran from which *Dunaliella salina* (a lake in Semnan Province) (34° 30′ 0” N, 51° 52′ 0″ E), *Dunaliella viridis* (Hovz‐e‐Sultan reservoir in Qom) (35° 0′ 27.01″ N, 50° 55′ 31.26″ E), and *Dunaliella* sp. (Bakhtegan lake in Fars province) (29° 21′ 55.44″ N, 53° 50′ 18.6″ E) were obtained.

Three species of *Dunaliella* microalga, native to Iran, isolated from three different regions (Figure 1), were obtained from Iran's National Center of Genetic Resources. The selected species were:

1. *Dunaliella salina* (IBRC‐M 50030), obtained from a lake in Semnan province (34° 30′ 0” N, 51° 52′ 0″ E)
2. *Dunaliella viridis* (IBRC‐M 50069), obtained from Hovz‐e‐Sultan reservoir (35° 0′ 27.01” N, 50° 55′ 31.26″ E)
3. Dunaliella species (IBRC‐M 50065), obtained from Bakhtegan lake in Fars province (29° 21′ 55.44” N, 53° 50′ 18.6″ E)

### 2.2 Culture conditions

The species were cultivated in the Janson culture medium, one of the most common culture mediums for *Dunaliella* species (Colusse et al., 2020). A five‐day‐old culture of green cells consisting of inoculum with 10% volume in 200 mL of the Janson liquid culture medium in 500 mL flasks was used and maintained at 4000 Lux light intensity, 12/12 h light/darkness and 25°C for 15 days. During these 15 days, sampling and examination of the samples were carried out on days 3, 5, 7, 9, 11, 13, and 15 (Pourkarimi et al., 2020).

### 2.3 Cell growth

The growth of the isolates was determined by measuring the optical density (OD) using a spectrophotometer (2100 model UNICO) at a wavelength of 680 nm (Zhu et al., 2018).

### 2.4 Dry biomass content

The microalgal dry weight (DW) was determined using a 50 mL culturing broth separated from the cultivated solutions. It was then filtered with a pre‐weighed Whatman GF/C filter (0.45 μm), and it is dried at 60°C for 24 h (Luo et al., 2021).

### 2.5 Protein content

Polyvinylpyrrolidone (0.05 g) and phosphate buffer (pH = 7) were mixed with 100 mL of the cultured microalgae cell set and centrifuged (Universal 320R made in Iran) at 9000*g* for 3 min at 30°C. The supernatant was mixed with 2 mL of Coomassie Blue dye, and then its absorbance was read with a spectrophotometer (2100 model UNICO) at a wavelength of 595 nm. The standard curve was prepared using bovine serum albumin (Bradford, 1976).

### 2.6 Carbohydrate content

The modified method of phenol and sulfuric acid was used to calculate the total carbohydrate (mono and di‐saccharide). The sample was mixed with phenol (5%) and then H_2_SO_4_ was added at 25°C. Its absorbance was read at the wavelength of 485 nm using a spectrophotometer (2100 model UNICO) based on the calibration curve and using standard glucose concentrations (Nielsen, 2010a).

### 2.7 Dietary fiber content

The dietary fiber content (hemicellulose) was calculated via the Van Soest method (Van Soest et al., 1991). A dry biomass sample was weighed, and an acid detergent fiber solution and a neutral detergent fiber solution were added to it. Boiling was adjusted to an even level and refluxed for 60 min, timed from onset to boiling. Then the samples were filtered, dried at 100°C for 8 h, and weighed. The percentage of fiber or cell wall constituents was calculated in the neutral detergent residual.

### 2.8 Total fat content

The amount of fat in the sample was measured using the Soxhlet method from dehumidified samples (Nielsen, 2010b). A dry biomass sample was placed in a Soxhlet extractor system (Iran Peco automatic Soxhlet machine) in petroleum ether for 6 h. The fat percentage of the biomass sample was calculated.

### 2.9 Fat extraction and determination of fatty acid profile by GC

Fat was extracted via the Bligh and Dyer method, and then the fatty acid compounds were measured using a TG 2552 spectrometer gas chromatography device with a flame‐ionization (FID) detector and an HP‐88 (Agilent Technologies, 100 m × 0.250 mm × 0.20) column (Bligh & Dyer, 1959; Xi et al., 2020).

#### 2.9.1 Nutritional value and healthy fat index assessment

These indices were obtained through the composition of fatty acids and the nutritional value of fat by using the Σn3:Σn6 ratio. Moreover, the atherogenic index (AI), thrombogenic index (TI), hypocholesterolemic/hypocholesterolemic (HH), and health‐promoting index (HPI) could be calculated by using Equations (1), (2), (3), (4) (Pekkoh et al., 2022; Šimat et al., 2015).

(1) $$\mathrm{AI}=\left(C12:0+4C14:0+C16:0\right)/\left(\sum \mathrm{UFA}\right)$$

(2) $$\begin{array}{cc} \mathrm{TI}=\; & \left(C14:0+C16:0+C18:0\right)/(0.5\sum \text{MUFAs}+0.5\sum n6\\ & -\text{PUFAs}+3\sum n3-\text{PUFAs}+\left(\sum n3/\sum n6\right) \end{array}$$

(3) $$\mathrm{HH}=\left(\mathrm{cis}-C18:1+\Sigma \text{PUFA}\right)/\left(C12:0+C14:0+C16:0\right)$$

(4) $$\mathrm{HPI}=\mathrm{\Sigma UFA}/\left[C12:0+\left(4\times C14:0\right)+C16:0\right]$$

### 2.10 Pigment content

Microalgal biomass (3 mL) was centrifuged at 800 *g* for 15 min. Aceton (3 mL) was mixed to wet the pellet for 20 s; finally, the mixture was centrifuged (Universal 320R) at 800 *g* for 10 min. The absorption quality of the upper clear solution was then read by the spectrophotometer (2100 model UNICO, China) for chlorophyll *a*, chlorophyll *b*, and carotenoids at 662 nm, 645 nm, and 470 nm wavelengths, respectively. Finally, total carotenoids and chlorophylls were calculated using Equations (5), (6), (7), (8) (Lichtenthaler & Buschmann, 2001; Schoefs, 2002).

(5) $$\text{Chla}\;\left(\mathrm{\mu g}/\mathrm{ml}\right)=11.24\times A_{662}-2.04\times A_{645}$$

(6) $$\text{Chlb}\;\left(\mathrm{\mu g}/\mathrm{ml}\right)=20.13\times A_{645}-4.19\times A_{662}$$

(7) $$T\;\mathrm{Chl}\;\left(\mathrm{\mu g}/\mathrm{ml}\right)=\text{Chla}+\text{Chlb}$$

(8) $$\text{Cart}\;\left(\mathrm{\mu g}/\mathrm{ml}\right)=\left(1000\times A_{470}-1.9\times \text{Chla}-63.14\times \text{Chlb}\right)/214$$

where Chla: chlorophyll *a*; Chlb: chlorophyll *b*; Chl: total chlorophyll; Cart: carotenoid.

### 2.11 DPPH radical‐scavenging assay

The antioxidant capacity of the used dry biomass was calculated by applying the 2,2‐diphenyl‐1‐1‐picrylhydrazyl (DPPH) method. Different concentrations of butylated hydroxytoluene (BHT) were then used to draw the standard curve (Singh et al., 2019).

### 2.12 Total phenolic compounds

Total phenolic content (TPC) was measured by the Folin–Ciocalteu colorimetric method with gallic acid as the standard. To describe briefly, the sample (1 mL) was extracted for 10 min at 25 ± 1°C with a 25 mL methanol solution (80% v/v). The extracted solution (0.5 mL) was mixed with 2.5 mL of folin reagent (10%). Then, sodium carbonate (2 mL, 7%) was added to this mixture. The mixture was obtained after shaking and further centrifuged (Universal 320R) at 10,000 rpm for 10 min; it was allowed to stand for 30 min in the dark, and absorbance was measured at 750 nm by using the UV–vis spectrophotometer (UV 2100, China). The calibration curve of gallic acid was used to calculate TPC. The obtained results were expressed as gallic acid equivalent (GAE) mg/100 g of the dry weight (DW) of the powder (Chen et al., 2015).

### 2.13 Statistical analysis

Statistical comparisons of the samples were performed using one‐way analysis of variance (ANOVA). Differences between the means were considered significant at the level of 5% (*p* < .05). The data were expressed as means ± standard deviations (SD) of three replicate determinations.

## 3 RESULTS AND DISCUSSION

### 3.1 Biomass growth rate

**FIGURE 2:**
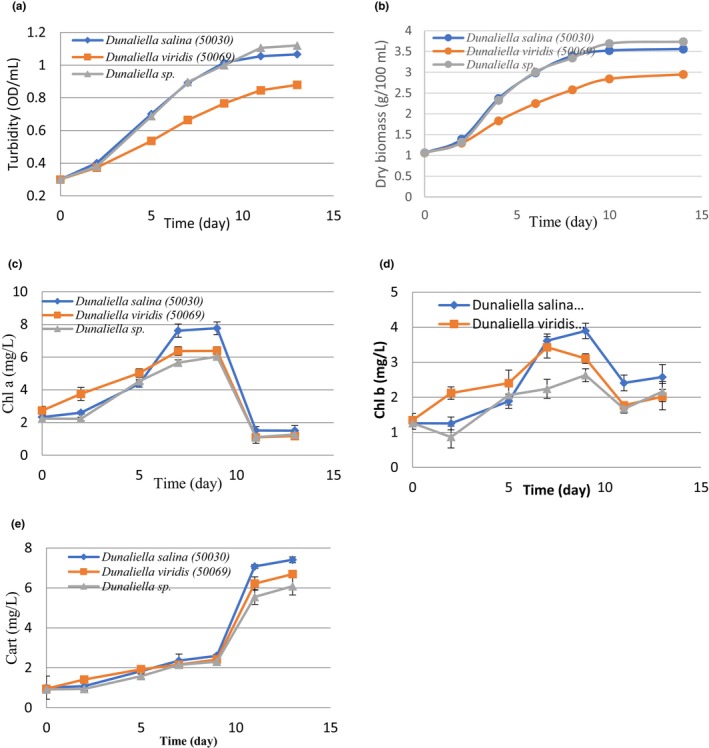
Growth changes as turbidity (OD/mL) (a), dry biomass (g/100 mL) (b), chlorophyll a (Chla) (c), chlorophyll b (Chlb), (d) and carotenoid content, (e) (mg/L biomass) of *Dunaliella salina* (50030), *Dunaliella viridis* (50069), and *Dunaliella* sp. (50065) during the incubation time (day) at 25°C.

The cell growth curve in the three isolates of *Dunaliella* (*Dunaliella salina*, *Dunaliella viridis*, and *Dunaliella* sp.), based on turbidity (OD/ml) and dry biomass (g/100 mL), is shown in Figure 2a,b. The results of the variance analysis indicated a significant effect of *Dunaliella* species and incubation time on the changes in growth (*p* < .05). Heterotrophic microalgae have three distinct growth phases: lag phase, exponential phase, and stationary phase. Photoautotrophic microalgae, such as *Dunaliella* species, have an additional feature called a linear growth phase, which is caused by the penetration of light into the dense algae culture medium (Aziz et al., 2020). Research has shown that the transition from the exponential phase to linear growth occurs only with light limitations (Schuurmans et al., 2017).

This stage can be postponed or prevented by changes in light intensity. The results showed that *Dunaliella* species were in the delayed phase until about the second day (Figure 2a,b). Changes in growth also had a higher slope in the early days until the 11th day and a lower slope angle from the 11th day onwards (Figure 2a,b). The intensity of biomass production on the beginning days was faster; later on, approaching the stationary phase, it had a lower slope (Yuan et al., 2020). Modeling of *Dunaliella* growth during 15 days of cultivation and changes in the rate of production slope also confirmed the results of the recent research (Yuan et al., 2020). In general, the amount of biomass produced and duration of the delay phase in the growth curve of microalgae can vary up to a few days, depending on the tolerance of the *Dunaliella* species against different environmental conditions, such as temperature, light intensity, salt content, pH, and nutrients in the culture medium (Colusse et al., 2020; Xi et al., 2020).

### 3.2 Pigment content

#### 3.2.1 Chlorophyll (chlorophyll a, b)

The trend of changes in the amount of total chlorophyll, chlorophyll *a* (Chla) and *b* (Chlb) was the same in all three isolates, as shown in Figure 2c,d. In the first 7 days of growth, the increase in the content of these pigments was significant (*p* < .05). However, the production of chlorophyll decreased after the 10th day; thus, no significant changes occurred, and the chlorophyll *a* and *b* content remained constant from day 12 to day 14.

Chlorophyll was one of the major pigments observed in all three isolates. It comprised 1%–5% of the algal dry weight. Chla was the most abundant pigment in *Dunaliella* (Figure 2c). Among the studied isolates, chlorophyll accumulation was significantly high in *Dunaliella salina* (50030) (*p* < .05). Chla is the main active phytochemical pigment in photosynthetic organisms (Biswal & Biswal, 1999). In many phytogenic groups, the light accumulation rate increases due to other active pigments (Wood, 1979). The most influential member of these pigments in green microalgae is Chlb. Among the studied isolates, Chlb accumulation varied from 2.01 to 2.58 mg/L, which was higher than Chla accumulation (Table 1).

**TABLE 1:** Pigment content (mg/L (biomas)) in *Dunaliella salina*, *Dunaliella* sp., and *Dunaliella viridis*.

Isolate	Profile pigment							
	Chla	Chlb	TChl	Cart	Chla/Chlb	Cart/Chla	Cart/Chlb	ß carotene
*Dunaliella salina*	1.51	2.58	4.09	7.41	0.58	4.90	2.87	5.28
*Dunaliella* sp.	1.17	2.01	3.19	6.69	0.58	5.70	3.31	4.76
*Dunaliella viridis*	1.26	2.16	3.42	6.08	0.55	4.81	2.81	4.95

Abbreviations: Cart, carotenoid; Chl, chlorophyll; Chla, Chlorophyll *a*; Chlb, Chlorophyll *b*; T Chl, total chlorophyll.

Chla/Chlb ratio in all three species ranged from 0.58 to 0.585. The evaluation of Chla/Chlb ration is a function of growth rate in nutrient‐limited cultures and irradiance in light‐limited and light‐sufficient cultures. At high light levels, photosynthetic organisms face photosynthetic damage, causing disruption in photosynthetic photoreactions, including photosystem 2 (Goericke & Montoya, 1998; Xu et al., 2016). In this research, due to the decrease in Chla, the Chla/Chlb ratio was lowered.

#### 3.2.2 Carotenoid content

The concentration of carotenoid in the three isolated *Dunaliella* sp. varied from 6.08 to 7.41 mg/L (Table 1). The carotenoid content of *Dunaliella salina* (50030) was significantly higher than that of *Dunaliella viridis* (50069) and *Dunaliella* sp. (50065). *Dunaliella salina* can have more carotenoid accumulation potential than other *Dunaliella* species (Ye et al., 2008). The observed fluctuations in the carotenoid content in the three isolates of *Dunaliella* during 14 days showed an increasing trend (Figure 2e). Carotenoid content changes increased with a gentler slope from the beginning to about the 9th day; then until the 11th day, it continued with a much sharper slope. From day 11 until day 14, the change rate was lowered significantly (Figure 2e). As a noteworthy point, when the carotenoid content increased, chlorophyll content decreased with a decreasing slope (Figure 2c,d). The Cart/Chla ratio was higher than that of Cart/Chlb in all isolates (Table 1). Carotenoid pigments protect photosynthesizing cells by scattering and absorbing light energy (Ahmed et al., 2014). When these microalgae face environmental stress, they increase carotenoids and dispose of singlet oxygen created by chromophores using sunlight absorption, and protect chlorophyll, fats, proteins, and DNA from oxidative reaction damage (Ahmed et al., 2014). The biochemical and physiological processes of *Dunaliella salina* make the evolutionary mechanisms of adaptation and metabolic regulation of carotenoid synthesis possible. This carotenogenesis process in *Dunaliella salina* makes it more tolerant of variable environmental conditions (Gallego‐Cartagena et al., 2019; Lamers et al., 2008). The accumulation of ß‐carotene in the three isolates was similar to the assessment of carotenoids and significantly higher in *Dunaliella salina* (Table 1).

### 3.3 Carbohydrate

The carbohydrate (mono‐ and di‐saccharide) content of *Dunaliella salina*, *Dunaliella* sp. and *Dunaliella viridis* was 12.37, 10.26 and 7.59 g/100 g, respectively (Table 2). Carbohydrate accumulation in *Dunaliella salina* was significantly higher than that of the others (*p* < .05). Microalgal carbohydrates are utilized in various biofuel production processes, such as bioethanol, biobutanol, and biohydrogen (Chen et al., 2013). Since carbohydrate levels in *Dunaliella* are lower than those in other microalgae (Table 2), this species is utilized less frequently in carbohydrate accumulation and biofuel production (Goswami et al., 2022).

**TABLE 2:** Protein, carbohydrate (mono and di‐saccharide), lipid, and dietary fiber content (as % DW) in *Dunaliella salina*, *Dunaliella* sp., *Dunaliella viridis*, and other green microalgae.

Isolates	Protein	Carbohydrate (mono‐ and di‐saccharide)	Lipid	Dietary fiber	Reference
*Dunaliella salina*	22.50 ± 0.22	12.37 ± 0.26	13.19 ± 0.26	48.82 ± 0.08	This study
*Dunaliella* sp.	17.68 ± 0.11	7.59 ± 0.39	25.02 ± 0.15	48.16 ± 0.13	This study
*Dunaliella viridis*	19.67 ± 0.15	10.26 ± 0.13	20.80 ± 0.45	42.10 ± 0.14	This study
*Dunaliella* sp.	19	8	42		(Gharajeh et al., 2020)
*Chlorella* Sp.	56	22	19		(Debnath et al., 2021)
*Dunaliella* sp.	49–57	4–40.2	6–18.2	–	(Hossain & Mahlia, 2019)
*Dunaliella salina*	5.4	27.9	9.1	–	(Efremenko et al., 2012)
*Dunaliella tertiolecta*	8.3–31.3	46.5–50.6	18.0–23.5	–	(Debnath et al., 2021)
*Chlorella vulgaris*	51–58	12–17	14–22	–	Becker (2007)
*Spirulina platensis*	50–65	8–63	4–9	–	(Hossain & Mahlia, 2019)
*Chlorella* sp.	60.6	–	12.8	13	(Abdelnour et al., 2019)
*Chlorella pyrenoidosa*	59.17	28.44	2.61	28.44	(Tudor et al., 2021)
*Arthrospira platensis*	67.85	13.98	0.76	4.20	(Tudor et al., 2021)
*Arthrospira platensis*	46.76	3.3	1.40	42.82	Molino et al. (2018)
*Dunaliella salina*	10.3	25.31	3.49	8.97	Molino et al. (2018)

### 3.4 Dietary fiber content

Microalgae contain high amounts (25%–75%) of dietary fibers, which are carbohydrates indigestible in humans' digestive system. The dietary fiber content in *Dunaliella salina* (48.82%) was significantly higher than that of *Dunaliella viridis* and *Dunaliella* sp., as shown in Table 2. The dietary fiber of the isolated *Dunaliella* was higher than that of *Chlorella pyrenoidosa* and *Arthrospira platensis* (Tudor et al., 2021). *Dunaliella* cells lack a rigid polysaccharide wall but instead possess a thin elastic plasma membrane and mucous surface coating, which are rich in fiber compounds with probiotic qualities. Due to the existence of these substances, their flexibility, and their ability to adjust to environmental conditions, *Dunaliella* is considered the most tolerant photosynthetic eukaryote (Ben‐Amotz, 2003; Molino et al., 2018).

### 3.5 Protein content

The protein content in *Dunaliella salina* (22.50 g/100 DW) and *Dunaliella viridis* (19.67 g/100 DW) was significantly higher than that of *Dunaliella* sp. (17.68 g/100 DW) (*p* < .05). As can be seen in Table 2, there is remarkable variability in protein contents within the *Dunaliella* genus. The isolated *Dunaliella* contained a protein content similar to that of chicken (24%), fish (24%), and peanuts (26%) (Janssen et al., 2022). However, the protein production of *Dunaliella* was less than that of *Spirulina* and *Chlorella* (Table 2). Despite the high protein content of *Spirulina* and *Chlorella*, direct utilization of its biomass is limited due to its single‐cell proteins' low digestibility and thick cell wall, while *Dunaliella salina*'s weak cell wall makes it a suitable and beneficial protein substance (Gong et al., 2014). Proteins, as an essential macronutrient for human growth, bring about good prospects for nutritional, medical, and pharmaceutical applications (Abomohra et al., 2016). Therefore, microalgal biomass production has been a promising method to compensate for the predicted long‐term protein shortage (Amorim et al., 2021).

### 3.6 DPPH radical‐scavenging activity

The antioxidant activity of the extracts was determined by applying the DPPH radical scavenger assay. According to the obtained results, as shown in Table 3, three isolated *Dunaliella* extracts had the ability to scavenge DPPH at various degrees (*p* ≤ .05). The antioxidant activity of the extracts of *Dunaliella salina*, *Dunaliella* sp., and *Dunaliella viridis* was 34.54, 30.37, and 26.91 mg/kg. In this regard, the inhibition percentage was higher than 50% in *Dunaliella salina* (Table 3). Thus, *Dunaliella salina* can be regarded as a rich source of antioxidants such as carotenoids, phenolic compounds, vitamins, and sulfated polysaccharides, which have a low molecular weight and a crucial role in the algae's antioxidant capacity and prohibit many oxidation processes or cell reactions to oxygen, peroxide, and free radicals (Cornish & Garbary, 2010; Gallego‐Cartagena et al., 2019). There are also many reports depicting the antioxidant properties of microalgae such as *Dunaliella*, *Spirulina*, and *Chlorella* (Goiris et al., 2012; Hajimahmoodi et al., 2010; Roy et al., 2021; Wu et al., 2005). *Dunaliella* can produce more antioxidants, such as carotenoid, to protect its photosystems' central reactions in the photosynthesis process when faced with stress conditions such as exposure, salinity, pH, temperature, nutrients, and nitrogen sources (Mishra & Jha, 2011) and accumulate them in their green tissues (Ye et al., 2008).

**TABLE 3:** Total phenolics, total antioxidant, and DPPH (% inhibition) in the microalgae biomass.

Species	Total phenolics (mg/100 g)*	Total antioxidant (mg/1000 g)**	%inhibition DPPH
*Dunaliella salina*	1.87 ± 0.01	34.54 ± 1.24	55.63 ± 2.03
*Dunaliella* sp.	1.68 ± 0.09	30.37 ± 0.66	47.97 ± 1.35
*Dunaliella viridis*	2.42 ± 0.02	26.91 ± 0.55	40.92 ± 0.68

*Note*: Values are given as mean (*n* = 3).

*As gallic acid equivalent.

**As BHT equivalent.

### 3.7 Total phenolic compounds

The total phenolic content (TPC) of *Dunaliella viridis* was significantly higher than the TPC of *Dunaliella salina* and *Dunaliella* sp. (*p* ≤ .05) (Table 2). Phenolic compounds are a group of phytochemical substances involved in various biologic activities, including antioxidant properties, anti‐inflammatory, and antimicrobial functions (Zarei et al., 2016). Phenolic compounds are also among the internal non‐enzymatic antioxidant systems in photosynthetic organisms that are able to reduce ROS production and inhibit it (Mishra & Jha, 2011; Ugya et al., 2019). The TPC of *Dunaliellas* obtained from Mraco's beaches was much higher than that of *Chlorella* (Maadane et al., 2015). The TPC of *Cholorellas* was between 0.75 and 2.21 mg/g GAE (Goiris et al., 2012). *Dunaliella salina*'s phenolic compounds in Safafar's research conditions were higher than the related results obtained in the recent research (Safafar et al., 2015). The TPC of microalgea, however, is dependent on the cultivation conditions and the methods of extraction. Moreover, the TPC in three microalga obtained from south‐east India showed that this substance was highest in *Chlorella*, *Dunaliella salina*, and *Navicula clavata*, respectivley (Hemalatha et al., 2013).

### 3.8 Total lipid

The lipid content of *Dunaliella salina*, *Dunaliella viridis*, and *Dunaliella* sp. was 13.19%, 20.80%, and 25.02%, respectively (Table 2). Research has shown the lipid content in different *Dunaliella* species varies from 6% to 71%, based on the dry biomass weight; the same quantities for *Chlorella* and *Spirulina* have been reported to be 14%–22% and 4%–9% (Hossain & Mahlia, 2019). The lipid content of the three isolated *Dunaliella* was in the middle of the data range for *Dunaliella* genus. *Dunaliella salina* ‘s lipid content in a recent study was higher than the similar results obtained from *Dunaliella salina* and *Chlorella* (Table 2) (Molino et al., 2018). Therefore, different lipid contents may be the result of differences in the type of species and environmental stress (Liu et al., 2013).

### 3.9 Fatty acid profile

The preliminary fatty acids of all isolated *Dunaliella salina*, *Dunaliella viridis*, and *Dunaliella* sp. revealed palmitic acid (16:0) (15.4%, 10%, and 11.2%), oleic acid (15.02%, 9.8%, and 10.36%), and α‐linolenic acid (23.35%, 10.15%, and 11.12%). The FA profile of *Dunaliella salina*, *Dunaliella viridis*, and *Dunaliella* sp. was close to that of the *Dunaliella* genus, as reported by Zonouzi et al. (2016).

In the FA profile of these three isolates of *Dunaliella*, the highest content of PUFA was observed in *Dunaliella salina*, while *Dunaliella viridis* and *Dunaliella* sp. showed the highest amount of SFA (Table 4). These results were, thus, in line with those of Maadane et al. (2015). However, SFA content in a kind of *Dunaliella salina* surpassed the PUFA one (Sahu et al., 2013). While docosahexaenoic acid (DHA) and eicosapentaenoic acid (EPA), two necessary fatty acids with many antioxidant properties, were seen in considerable amounts in the three isolated *Dunaliella*, their content was the highest in *Dunaliella salina* (Table 4). Therefore, it is recommended to analyze the fatty acid profile in these microalgae after changes are applied in cultivation conditions and the nutrients in future studies; this is because *Dunaliella* species contain long‐chain polyunsaturated acids with high antioxidant properties (Maadane et al., 2015; Talebi et al., 2013).

**TABLE 4:** Fatty acid profile of *Dunaliella salina*, *Dunaliella* sp., *Dunaliella viridis*, and other microalgae.

Species	EPA (%)	DHA (%)	UFA:SFA	Σn3:Σn6	⅀n6 (PUFA)	⅀n3 (PUFA)	⅀PUFA	⅀MUFA	⅀SFA	Reference
*Dunaliella salina*	11.26	6.15	1.77	2.79	10.59	29.63	40.13	23.80	35.99	This study
*Dunaliella* sp.	6.85	4.91	0.90	1.37	10.71	14.70	25.42	22.02	52.48	This study
*Dunaliella viridis*	4.91	5.14	1.03	1.00	15.24	15.28	30.53	20.36	49.03	This study
*Dunaliella* sp.	1.4	0.6	1.2	3.6	9	31.9	45.4	7.8	46.3	Gharajeh et al. (2020)
*Dunaliella salina*	–	15.4	1.9	–	–	–	33.1	33.2	34.8	(Fakhry & Maghraby, 2013)
*Dunaliella salina*	21.4	–	0.68	–	–	–	24	16.1	59.2	Bhosale et al. (2010)
*Chlorella* sp.	–	3.24	1.02	0.81	15.3	12.9	28.8	24.4	49.9	Sahu et al. (2013)
*Desmodesmus* sp.	–	–	0.162	–	–	–	57.59	28.4	13.98	(Ferreira et al., 2019)
*Picochlorum* sp. *SBL2*	–	–	2.4	–	–	–	36.62	34.01	29.37	Pereira et al. (2013)
*Spirulina platensis*	0.19	–	0.74	–	–	–	30.17	11.20	55.72	(Ötleş & Pire, 2001)
*Chlorella vulgaris*	–	0.30	2.63	–	–	–	38.30	28.04	25.26	(Ötleş & Pire, 2001)

The Σn3:Σn6 ratio of the three isolated *Dunaliella* varied from 1.00 in *Dunaliella viridis* to 2.79 in *Dunaliella salina*. This significant ratio showed the high nutritional value of fats in these species. Furthermore, the Σn3:Σn6 ratio, recommended by the World Health Organization (WHO), is more than 1.5; meanwhile, in the case of many different fishes, this value has been reported to be less than two (Muehlroth et al., 2013). Consuming foods with a certain ratio of fatty acids contributes to a healthy life. The ratio of Σn3:Σn6 fatty acids is considered a usable index for oleaginous food. Research has shown that the balance between n3 and n6 can preserve human health, and improper and unbalanced consumption of n3 and n6 can cause cancer, chronic neuro‐diabetes, cardiovascular diseases, and inflammatory diseases. The balance between their ratios can reduce the dosage of medicine in some diseases (Rubio‐Rodríguez et al., 2010). The recent study, thus, proved that the three isolated *Dunaliella* fatty acids had this balance, making these microalgae highly beneficial to human health.

### 3.10 Nutritional value and healthy fat index assessment

Atherogenic index (AI) and thrombogenic index (TI) can be applied to examine the atherogenic and thrombogenic potential of fatty acids. AI shows the correlation between the total of saturated fatty acids and that of unsaturated fatty acids; thus, consuming food with lower AI can reduce the level of total cholesterol and low‐density lipoprotein cholesterol in the human blood plasma. TI is the correlation between fatty acids that cause thrombogenesis (C12:0, C14:0, and C16:0) and anti‐thrombogenic fatty acids (Σn3, Σn6, MUFA); thus, foods with lower TI are beneficial for cardiovascular health (Chen & Liu, 2020). The hypocholesterolemic index (HH) can be considered to evaluate the effects of combining fatty acids on cholesterol and determe the correlation between hypocholesterolemia and hypercholesterolemic fatty acids. The high ratio, along with low AI and TI, plays an important role in reducing the incidence of coronary heart diseases. The health‐promoting index (HPI) is utilized to evaluate the nutritional value of dietary fat, which relates to the composition of fatty acids and cardiovascular diseases (Kent et al., 2015).

So, the lower the AI and TI of the oily product, the healthier the food source (Chen & Liu, 2020). The AI and TI of the three isolated *Dunaliella* varied from 0.47 to 0.59 and from 0.19 to 0.25, respectively. AI and TI of *Dunaliella salina* had the lowest values, which was more advantageous when compared to food candidate microorganisms such as *Spirulina* sp., *Chlorococcum amblystomati* (Conde et al., 2021), and *Anomoeoneis* sp. (Pekkoh et al., 2022) (Table 5).

**TABLE 5:** Atherogenic index (AI), thrombogenic index (TI), hypocholesterolemic (HH), and health‐promoting index (HPI) health indices of the fatty acid profile in *Dunaliella salina*, *Dunaliella* sp., *Dunaliella viridis*, and the nutritional candidate microalgae.

Health indices	AI	TI	HH	HPI	Reference
*Dunaliella salina*	0.47	0.19	2.90	2.11	This study
*Dunalliella* sp.	0.55	0.25	2.97	1.82	This study
*Dunalliella viridis*	0.59	0.23	2.334	1.70	This study
*Anomoeoneis* sp.	0.68	1.15	0.57	1.46	Pekkoh et al. (2022)
*Dunaliella* sp.	0.63	0.42	1.85	–	Gharajeh et al. (2020)
*Chlorella vulgaris*	0.38	0.27	2.04	–	(Jahromi et al., 2022)
*Spirulina* sp.	0.7	1.6	0.6	–	Conde et al. (2021)
*Scenedesmus obliquus*	0.2	0.1	2.8	–	Conde et al. (2021)
*Chlorococcum amblystomatis*	0.5	0.3	1.4		Conde et al. (2021)

HH and HPI were significant in the three isolates, and both indices were optimal, especially in *Dunaliella salina*. Examining these indicators showed that the composition of fatty acids in these three species, especially *Dunaliella salina*, not only contributed to reducing the incidence of diseases such as coronary heart disease, but also had a high nutritional value.

## 4 CONCLUSION

Our investigation of the natural chemical and bioactive compounds of the three native isolated microalgae, namely, *Dunaliella salina*, *Dunaliella viridis*, and *Dunaliella* sp., showed promising results. These three types of biomass contained substantial amounts of protein, dietary fiber, suitable carbohydrates, and high‐quality fats rich in PUFA, EPA, and DHA. Furthermore, the health indicators (AI, TI, HH, and HPI) obtained from the fatty acid profile, especially in the case of *Dunaliella salina*, proved the high nutritional value of the fats in these species. Since humans, animals, and many higher plants lack the necessary enzymes for the synthesis of omega‐3 fatty acids, they must receive them from external sources. These three types of microalgae, especially *Dunaliella salina*, can be good sources of this fatty acid. EPA and DHA fatty acids can be commercialized from these microalgae and used as food additives, pharmaceuticals, cosmetics, and baby food. The accumulation of antioxidant pigments, such as beta‐carotene, especially in *Dunaliella salina* (IBRC‐M 50030), as well as the presence of bioactive phenolic compounds, in this microalga can turn it into a rich source of antioxidants. *Dunaliella salina*, *Dunaliella viridis*, and *Dunaliella* sp. have the potential to be used in the food and pharmaceutical industries, to produce highly nutraceutical and functional foods and enhance human health in society.

Our findings, thus, highlight the unique biochemical and bioactive components of these microalgae, and prove that they, especially *Dunaliella salina*, can be used as a natural cell factory for the biological synthesis of bioactive substances and a new and sustainable source for human food needs. At the same time, these features, especially valuable polyunsaturated acids, EPA, DHA, and antioxidant pigments, make these species superior to *Chlorella* and *Spirulina*. Optimizing the growth conditions of these species, especially *Dunaliella salina* (IBRC‐M 50030), which originates from the lake in Semnan province (34° 30′ 0” N, 51° 52′ 0″ E), Iran, is recommended to increase the accumulation of biomass and natural bioactive compounds. They can be used as whole or cracked cells to enrich food or produce new food products, along with technical and economic evaluations, which can be recommended for future studies.

## AUTHOR CONTRIBUTIONS

**Maryam Araj‐Shirvani:** Conceptualization (equal); data curation (equal); formal analysis (equal); investigation (equal); methodology (equal); project administration (equal); resources (equal); software (equal); validation (equal); visualization (equal); writing – original draft (equal); writing – review and editing (equal). **Masoud Honarvar:** Conceptualization (equal); data curation (equal); formal analysis (equal); investigation (equal); methodology (equal); project administration (equal); resources (equal); software (equal); supervision (equal); validation (equal); visualization (equal); writing – original draft (equal); writing – review and editing (equal). **Mahshid Jahadi:** Conceptualization (equal); data curation (equal); formal analysis (equal); investigation (equal); methodology (equal); project administration (equal); resources (equal); software (equal); supervision (equal); validation (equal); visualization (equal); writing – original draft (equal); writing – review and editing (equal). **Maryam Mizani:** Conceptualization (equal); data curation (equal); formal analysis (equal); investigation (equal); methodology (equal); project administration (equal); resources (equal); software (equal); supervision (equal); validation (equal); visualization (equal); writing – original draft (equal); writing – review and editing (equal).

## CONFLICT OF INTEREST STATEMENT

The authors declare that they do not have any conflicts of interest.

## ETHICS STATEMENT

This study did not involve any human or animal testing.

## INFORMED CONSENT

Written informed consent was obtained from all study participants.

## Data Availability

The data that support the findings of this study are not shared.
